# Obesity as a Risk Factor in the Appearance of Haematomas Caused by Low-Molecular-Weight Heparin: A Cross-Sectional Study

**DOI:** 10.3390/nursrep13020067

**Published:** 2023-05-01

**Authors:** Candelaria de la Merced Díaz-González

**Affiliations:** Department of Nursing, Faculty of Health Sciences, University of Las Palmas de Gran Canaria, Juan de Quesada, 30, 35001 Las Palmas de Gran Canaria, Spain; candelaria.diazg@ulpgc.es

**Keywords:** thromboembolic disease, low-molecular-weight heparin, obesity, haematoma, skinfold

## Abstract

Thromboembolic disease (TED) is an important health problem in Europe due to its high morbimortality. Pharmacological prevention is achieved with low-molecular-weight heparin (LMWH), among other strategies, which are supported by a high degree of evidence in the scientific literature. According to its safety data sheet, this injection produces local injuries at a rate of 0.1–1% after administration; however, these percentages are much lower than others reported in several studies focusing on LMWH (44–88%). This high incidence of injuries might be associated with procedural or individual variables. (1) Background: Among the most frequent side effects after the administration of LMWH are pain and haematomas (HMTs), which are influenced by obesity. We aimed to determine the relationship between abdominal skinfold (ASF) value and incidence of HMTs. In addition, I sought to determine how the risk of HMT changed with each mm increase in ASF. (2) Methods: A cross-sectional descriptive study developed in the hospital unit of orthopaedic and trauma surgery was conducted over one year. All participants in the sample were classified based on their ASF and the appearance and area of HMTs were assessed after the administration of enoxaparin. The STROBE checklist was used to evaluate the study. Descriptive statistical analysis and analysis of variance of non-parametric factors were carried out. (3) Results: In a sample of 202 participants (808 Clexane injections), more than 80% presented HMTs. More than 70% of the sample was overweight and more than 50% had an ASF > 36 millimetres (mm). (4) Conclusions: An ASF over 36 mm confers a higher risk of developing HMTs: with each mm increase in the ASF, the risk increases by 4%. Participants who are overweight or obese also present a higher risk of HMT, and these conditions correlate positively with the area of the HMTs. Providing education for the self-administration of the drug after discharge and information about the probability of suffering from local injuries in a more individualised way will lead to fewer primary care nursing consultations, more adherence to the antithrombotic treatment, and, as a consequence, a decrease in TED and health costs.

## 1. Introduction

Subcutaneous (SC) administration of low-molecular-weight heparin (LMWH) is performed daily by a nurse in the hospital according to the medical prescription. Throughout the more than 40 years of use of this drug, the literature has revealed many variables in the administration procedure that have changed over time. Some of these variables have now been eliminated due to the use of pre-filled syringes, but many others continue to change. The literature also mentions possible adverse reactions [1,2]. Specifically, the safety data sheet for enoxaparin (Spanish Agency for Medicines and Health Products [AEMPS]) [3] cites the following adverse reactions associated with its administration: urticaria, pruritus, erythema, pain, bruising, bleeding, hypersensitivity, and oedema, among others. Focusing on local reactions in the injection area, the aforementioned file reports an incidence of 0.1–1% for moderate local irritation, pain, bruising, and ecchymosis and, on rare occasions, hard lumps (0.01–0.1%). However, the literature from the perspective of nursing care and focused on the drug administration procedure [4,5,6,7,8,9,10,11] reports a higher incidence of local lesions in the injection area and contradicts previous data. Local haematomas (HMTs) can occur at a rate of 40–88% [4,12], depending on the variables modified in the administration procedure.

In the literature on the administration technique of LMWH by nurses, the first references date from 1981 [13], and since then, its evolution has been taken up in both nursing manuals and scientific articles. In each of them, each of the phases of the procedure is described: verification of the prescription, verification of the product to be administered and dose, identification of the patient, preparation of the drug, selection of the area where the drug will be administered, cleaning of the selected area, instructions for during the injection technique, instructions for immediately after the injection, monitoring of the area, and registration of the procedure carried out by nurses.

LMWH has several secondary effects. A multitude of studies [4,5,6,7,8,9] reported this and searched for modifications to the administration technique to reduce or eliminate these effects. This suggests that these effects may be the sum of two factors: the effect of the drug and the administration technique used by the healthcare professional.

Different authors [4,5,6,7,8,9] carried out studies modifying variables involved in the administration technique to find modifications that might reduce pain, itch, and local HMTs. Among the variables tested in these studies are age, gender, pathology, type of LMWH, disinfect the area before injection, interior diameter and length of the needle, area selected for the injection, method of holding the skin (fold) during the injection, time or speed of injection, mode of extraction, pressure on the injection area afterwards, cleaning technique after injection, and monitoring.

The literature [4,5,6,7,8,9,14,15,16,17,18,19,20,21,22,23] shows high incidence of HTMs, of up to 88.9% [15]. Several authors included anthropometric variables in their studies, relating these to the appearance of HMTs, and showing that obese participants had a higher rate of HMTs [7,8,14], also finding a positive correlation between the non-appearance of HMTs and the elimination of HMTs of the pinch (skin fold formation) before injecting the obese participant [7].

Nurses must cross the skin barrier when administering LMWH using an invasive technique [24] to place the drug under the skin (into the SC tissue, where absorption of the product occurs through simple diffusion into blood vessels. To reach this tissue, it is necessary to use a needle to go through the epidermis, which consists of five layers, then the dermis, where the presence of a tortuous vascular network makes it difficult to pass through without damaging any of them (such as the papillary loops, superficial vascular plexus, and deep capillary vascular plexus) and finally reaching the SC tissue, where the SC vascular plexus can also rupture if touched and spill its contents to the outside, creating a HMT [25,26,27,28,29,30,31]. Several authors [32,33,34] affirmed that the greater number of blood vessels in women and the action of oestrogens produce an increase in the vascularisation of the area and result in greater bleeding [34]. Furthermore, during ageing, the blood vessels of the dermis become more fragile, resulting in their being easily injured.

It is necessary to consider the increase in the prevalence of obesity in Europe (15.7%) and the overweight populations (37.7%) [35] and look for LMWH administration procedures for this group that reduce the rate of HMTs.

The most widely used anthropometric parameters [36,37,38] to assess the nutritional status of patients are weight, height, perimeters, diameters, skinfold (SF), and BMI. The World Health Organization (WHO) [39] classifies obesity based on BMI, emphasizing categories, and within the category of obesity (BMI > 30), there are three subcategories (obesity I, II, and III). The SF are anthropometric indicators, and each SF consists of a double layer of skin and SC adipose tissue. SF measurement is a rapid and non-invasive procedure [24,40], although it is susceptible to errors due to variability in SC fat compressibility, and the larger the folds, the more difficult it is to perform [28]. When using folds, one should keep in mind that total body fat increases with age, although SF may not change because fat accumulates largely in different locations than SC fat [24]. One of the most used is the ASF, which is used for the administration of drugs in the SC tissue in this study.

A review of the literature on aspects that interfere with the administration of LMWH and adverse reactions has helped to identify the necessary keys to develop this study and assess the incidence of abdominal HMTs secondary to the administration of LMWH, as well as find a relationship between obesity (based on ASF and BMI) and both the incidence of the appearance of abdominal cutaneous HMTs and their area.

Scientific research in this field is justified for the following reasons: the need to determine the independent variables that influence the appearance of abdominal HMTs with the standardised injection technique, in addition to allowing the detection of possible risk groups that may be candidates for a technique of modified injection to reduce local lesions such as bruising, as this may have clinical benefit and improve adherence to treatment.

The objectives of this study were to identify the prevalence of abdominal HMTs secondary to administration to LMWH (Clexane), and to analyse the relationship between obesity (as determined by ASF and BMI) and the incidence of the appearance of abdominal cutaneous HMTs, as well as their location, in patients receiving Clexane.

## 2. Materials and Methods

We followed the strengthening the reporting of observational studies in epidemiology (STROBE) [41] recommendations in this study.

### 2.1. Design and Context

This is a cross-sectional descriptive study.

### 2.2. Setting

This study was developed and conducted in the hospital unit of orthopaedic and trauma surgery (HUOTS) at the Hospital Insular de Gran Canaria (HIGC) over one year, allow for the recruitment and evaluation of participants.

### 2.3. Participants

The participants in the study were among the patients admitted to the HUOTS during the study period. The inclusion criteria were being over 18 years of age, undergoing anticoagulant treatment with LMWH (only Clexane^®^), no bleeding disorder, absence of a diagnosis of mental disorders (Alzheimer disease, delirium, dementia) or temporary disorientation, and providing written informed consent to participate in the study.

The following selection process was carried out: (1) daily review of the new admissions in the unit (from the previous day); (2) accessing the electronic medical records (EMR); (3) verifying that the patient did not have any type of diagnosed cognitive impairment or temporary disorientation; (4) finding the treatment prescribed by the traumatologist and verifying the presence of Clexane; (5) going to the patient’s room and presenting the information sheet, data protection form and informed consent; and (6) obtaining consent from the patient, allowing their inclusion in the sample of participants.

### 2.4. Variables

The variables included in this study are presented in Table 1 and include sociodemographic, independent, and dependent variables.

### 2.5. Data Sources and Measurement

The procedure that is always carried out by the HUOTS nurses is as follows: (1) check the prescription; (2) inform the patient of the medication to be injected; (3) provide the patient with a quiet and private environment; (4) select the injection site (alternating right or left hemiabdomen); (5) clean the area and form the SF; (6) insert the needle, forming a 90 degree angle with the skin surface (this should be performed relatively quickly and without moving the needle); (7) keep the SF tight until the removal of the needle; (8) inject the medication (around 10 s); (9) remove the needle quickly and in the same direction from which it entered without moving it; (10) leave the cotton or gauze if there is blood loss while applying slight pressure; (11) explain the importance of avoiding massaging the area; and (12) record the medication administration in the patient’s EMR.

The evaluation tools used for data collection were: (1) EMR: provides the researcher with information on compliance with the inclusion criteria and data on the variables included in the study, such as gender and age; (2) scale with height rod: used to weigh (up to 130 kg) and measure (200 cm) the participant. It was calibrated by the HIGC electromedical department before the study; (3) BMI [37]: obtained using a formula that requires weight (kg) and height (m) for its calculation; (4) plicometer [42]: the plicometer or lipocaliper is a manual tool used to measure in millimetres (mm) the quantity of SC adipose tissue that the participant has by measuring the SFs. It has a range of 0–48 mm, a pressure of 10 g/m^2^, an accuracy of 0.2 mm, and a graduation of 0.2 mm; (5) syringe of LMWH [3]: pre-filled syringe with a single dose of enoxaparin at the commercial doses 20 mg, 40 mg, 60 mg, 80 mg, or 100 mg. This syringe is provided in a single-dose plastic case and comprises a cap, plunger, barrel, and 27 G needle (0.4 mm) with a length of 4/10 inch (1.016 cm) that cannot be separated from the barrel. The quantity of the drug in mL is proportional to the dose in mg (e.g., enoxaparin 0.2 mL = 20 mg). Enoxaparin was chosen for the present study because it is the only LMWH that the pharmacy service currently provides (under the trade name Clexane). The pharmacy service distributes fondaparinux sodium, but it is catalogued as a selective antithrombotic, not included among the LMWHs, and is currently used infrequently in the HUOTS; (6) marker: a black fine-tip permanent marker to mark the injection area of the day with four dots at cardinal points 1 cm from the injection site to enable the assessment of injuries in the area.

Measurement procedure: (1) on the day of recruitment of the participant, the ASF measurement was performed (three measurements were taken and the average was calculated); (2) the researcher witnessed one of the five nurses administering the injection; (3) the injected area was indicated with a marker (abdominal); (4) the injected area was reviewed at 24, 48, and 72 h, recording the presence or absence of HMTs; (5) the bruise was measured (length × width); and (6) each patient received 4 injections, on consecutive days.

### 2.6. Bias

The reliability of the measurements depends on the selection of tools with low variability and the calibration of tools that can be calibrated. In addition, validity of the measurements is ensured because each device was used for the purpose for which it was created (scale with height rod, plicometer).

To reduce bias, the tools were calibrated, and measurements were performed three times for each variable, calculating the average to provide a more accurate estimate. In addition, ASF measurements were performed before the administration of LMWH. Moreover, nurses avoided stimulating the injection site before and did not massage after the injection, as this could lead to the appearance of HMTs.

The researcher in charge of data collection was trained in the use of the lipocaliper to measure ASF and in the delineation measurement of abdominal lesions.

### 2.7. Study Size

The minimum sample size was 167 participants based on a 50% response distribution and a 95% confidence interval, in the GRANMO^®^ calculator (Institut Municipal d’Investigació Medica, Barcelona, Spain, Version 7.12).

To select the participants in this study, the following inclusion criteria were established: patients hospitalised in the HUOTS of the HIGC, absence of mental disorders, written consent to participate in the study, over 18 years of age, and on anticoagulant treatment with LMWH (Enoxaparin).

### 2.8. Statistical Methods

After data collection and measurements were carried out, they were registered in a database created for this purpose, which was statistically analysed with the statistical software Statistical Package for the Social Sciences (IBM, Armonk, NY, USA), with appropriate licensing, and R-Project version 3.1.0 (R Development Core Team, Vienna, Austria), which is free software. The following statistical analyses were carried out: descriptive statistical analysis and analysis of variance of non-parametric factors, Spearman’s rank correlation coefficient, and logistic regression for the estimation of crude and adjusted odds ratios (COR and AOR, respectively).

## 3. Results

### 3.1. Sample

The total number of participants was 202, and the sample number was valid for the analysis of variance of non-parametric factors and COR and AOR. Some missing data in some variables were attributed to chance, due to the absence of patients admitted with extreme obesity or very thin patients with ASF I, the year the study was undertaken.

### 3.2. Sample Characteristics

The average age of 64.39 years (range 32–89; standard deviation (SD) 15.04). The distribution by gender was 126 (62.4%) women and 76 (37.6%) men. The largest age group, comprising 49.5% of the participants, was 60–79 years.

### 3.3. Outcome Data

Regarding the diagnosis of the participants at admission, hip fracture was the most common diagnosis, constituting 23.8% of the sample, followed by coxarthrosis, with a little more than 10.4%.

All participants in the sample were prescribed pharmaceutical antithrombotic prophylaxis with LMWH, thus, meeting the inclusion criteria. Regarding the daily dose of LMWH, 183 participants (90.6%) were prescribed 40 mg of Clexane per day, 6 (3%) were receiving 60 mg, 9 (4.5%) were receiving 80 mg, and 4 (2%) were receiving 160 mg.

The incidence and characteristics of the HMTs in the 202 participants across all administered injections (n = 808) are shown below; the administration procedure used by the nurses in the unit where the study was carried out is described in the Materials and Methods. Regarding abdominal injuries after the application of the antithrombotic prophylaxis, 171 (84.7%) participants in the sample showed abdominal injuries in the form of HMTs, while a small percentage (15.3%; 31 participants) did not present cutaneous injuries. The distribution of HMTs in the participants (n = 202) was as follows: 31 (15.3%) presented no HMTs, 29 (14.4%) presented just one HMT from four administered injections, 64 (31.7%) presented two HMTs, 51 (25.2%) had three HMTs, and 27 (13.4%) presented four HMTs.

Of 808 administered injections (each participant receives four injections), 418 (51.7%) produced local injuries in the form of HMTs. The average number of HMTs on the right hemiabdomen was 2.16 (range 1–4), while on the left hemiabdomen it was 1.83 (range 0–3), with an average total of 2.07 HMTs (range 0–4; the sum of the HMTs located on the left and right hemiabdomen). Among the 202 participants, 62.6% of those who presented HMTs were women. This trend was echoed when considering the 808 injections, as 66.7% of the injections that caused HMTs were in women.

Among the characteristics considered for the HMTs, the area of the HMTs stands out. Among the total of 418 injuries identified as HMTs, the average area was 300.40 mm^2^ (range 0–3370; 0.465 in^2^). The average area of the injuries on the right hemiabdomen was 320.33 mm^2^ (range 0–2210) and on the left hemiabdomen was 279.39 mm^2^ (range 0–2620).

The area of the HMTs according to gender can be found in Table 2. The average area per participants with HMTs in the sample is more than 734.32 mm^2^ (1.138 in^2^). It is noteworthy that the average area was 786.57 mm^2^ in women and 646.97 mm^2^ in men. When comparing the average area of HMTs caused by the 808 injections (n = 418), the average for women was slightly higher (by approx. 3 mm^2^) than for men (301.6 mm^2^ vs. 297.9 mm^2^).

In Table 3, it is noteworthy that the youngest group (<40 years old) have the highest average area of HMTs among participants with injuries, followed very closely by the septuagenarian group. This is also observed when considering the average area of total injuries. This might be related to the small size of the youngest age group. However, when considering the average area of HMTs across the entire sample (n = 202), the age group with the highest number of participants (70–79 years) is the group that has the largest total area of HMTs by far (1020.6 mm^2^; 1.582 in^2^).

The average body weight of the participants in the sample was 79.53 kg (range 45.4–118, SD 17.93) and the average height was 165.58 cm (range 150–194, SD 9.72). The average abdominal perimeter was 100.35 cm (range 72–130, SD 14.93).

According to the participants’ BMIs, obesity was represented in the sample, as the average BMI was 28.81 (SD 5.08) and the range was 19.45–39.75. The distribution of the sample in the obesity classifications based on the BMI according to the WHO shows: underweight (<18.5) 0%; normal weight (18.5–24.9) 27.8%; overweight (25–29.9) 29.7%; type I obesity (30–34.9) 36.1%; type II obesity (35–40) 6.4%; extreme obesity ≥ 40 0%.

Based on the measurement of ASFs described in the Materials and Methods, the ASF values were categorised into quartiles that were interpreted as low, moderate, high, and very high. Quartile I—low (0–11.9) 3%; quartile II—moderate (12–23.9) 24.8%; quartile III—high (24–35.9) 28.2%; quartile IV—very high (36–48) 44.1%.

Table 4 presents the number of HMTs based on BMI and gender of the participants and shows that a plurality of HMTs (40.2%) occurred in the type II obesity group.

It can be observed that in the total sample, the SF IV represents more than 50% of the total HMTs, highlighting the highest percentage in the female gender. The HMTs data according to the ASF quartiles are represented in Table 5, where it can be seen that the highest ASF quartiles present a higher percentage of HMTs.

Table 6, which presents the distribution of abdominal HMT area according to ASF quartile, reveals that the largest average HMT area is found in SF II, where the average lesion reaches 452.9 mm^2^ (0.702 in^2^).

### 3.4. Main Results

To assess the relationship between ASF and the appearance of HMTs, the linear regression carried out in Table 7 was reviewed, revealing a significant relationship between the quartile ASF IV and the appearance of HMTs. It is also observed that a participant with an ASF over 36 mm has a 2.5 times (2.586; *p* < 0.001) greater risk of developing HMTs compared to individuals with a smaller ASF. A significant relationship is also observed when considering the AOR (which has been adjusted for age, gender, height, and abdominal circumference variables) associated with the presence or absence of HMTs, which reveals that a participant with an ASF > 36 mm has a 1.8 times (*p* < 0.05) greater risk of developing HMTs. A significant relationship (*p* < 0.001) also exists if we evaluate the ASF as a continuous variable, which establishes that for each mm that the value of the SF increases, the risk of developing HMTs increases 4%.

The relationship between the area of the HMTs and the ASF is shown in Table 7, which shows that the HMT area correlates significantly (*p* < 0.001) with the size of the ASF. However, if this relationship is adjusted by eliminating the variables age, weight, and height, the correlation no longer appears to be significant. Thus, it does not seem that the value of the ASF has any relationship with the area of the HMT if those confounding variables are eliminated. The distribution of the abdominal area of the HMTs (mm2) according to the BMI (WHO obesity criteria) shows in the case of the total area (mm2) in the participants (n = 171): normal weight 27,410 mm^2^; overweight 39,135 mm^2^; obesity type I 44,651 mm^2^; obesity type II 14,373 mm^2^. The average area (mm2) per participants (n = 171): normal weight 668.53 mm^2^; overweight 738.39 mm^2^; obesity type I 666.43 mm^2^; obesity type II 1437.3 mm^2^.

The frequency and percentage of HMTs based on the BMI category according to the WHO are shown below in Table 4 for the 418 HMTs that developed after the 808 injections. It is noteworthy that more than 40% of the HMTs developed in participants in obesity class I. This distribution is maintained for men, but for women, the HMTs are more dispersed among the different BMI categories.

In searching for a relationship between obesity according to the BMI categories and the appearance of HMTs in Table 7, COR shows that there is such a relationship (*p* < 0.05), with a 1.65 times (65%) greater risk of developing HMTs in overweight participants compared to participants in the normal weight category. This relationship also exists when using the AOR, which reveals a 1.56 times (56%) greater risk, or a slightly lower increase in risk among obese individuals.

The total and average areas of the HMTs, including only participants who developed HMTs (n = 171), based on the BMI was determined according to the criteria of the WHO. The obesity class II group has the highest average area of HMTs, followed by the overweight group; however, it is necessary to mention that the category obesity class II comprised only 10 participants.

To determine if there was any relationship between obesity based on BMI and the area of the HMTs, it was necessary to re-examine the results, which revealed a significant positive correlation (*p* < 0.001) between these variables. This relationship is similar to that found between the area of the HMTs and the ASF.

## 4. Discussion

In line with the objectives of this study, we analysed the influences of the participant characteristics examined on the incidence of HMTs following the administration of LMWH.

### 4.1. Characteristics of the Sample

The average age of the sample was 64.39 years. Notably, this figure is higher than that reported in studies [43] carried out in Spain in units with similar characteristics, in which the average age was 61 years.

Most (62.4%) of the sample consisted of women, as was reported in other studies [5,15,43,44]. Regarding the pathology at admission, the most common diagnosis in this study was hip fracture (23.8%). According to the Institute of Health Information [45], in Spain, the national rate of this pathology is 103.7/100,000 inhabitants, but the rate in the Canarian community is half the national rate (51.76). Understanding the causes of hip fracture is of growing importance for society at both the economic and social levels [45]. The sum of the diagnoses related to the knee (gonarthrosis, knee replacement implant, and infection of knee implant) that lead to the surgical placement of a knee implant, whether for the first time or repeat surgery, comprises 14.8% of the admission diagnoses of the sample of this study. Data published by the Spanish Society of Rheumatology [46] affirm that 10.35% of the population suffers from knee arthrosis, and the prevalence is very similar (10.17%) in the Canaries. The incidence of surgery for these conditions is increasing due to the ageing population and the unchanging relationship between age and arthrosis [47].

### 4.2. Antithrombotic Prophylaxis

Compliance with the antithrombotic prophylaxis with LMWH in this study was 100%, since having been prescribed this drug was an essential requirement for inclusion in the study. However, it is important to emphasise that all the participants who were initially selected because of their risk of TED (before confirming their participation in the study) were prescribed LMWH. Previous studies carried out in Spain that focused on LMWH reached 96% compliance [48]. Similarly, in the unit where the present study was developed, the guidelines [49,50,51,52] regarding pharmacological antithrombotic prophylaxis are fulfilled.

### 4.3. Relationships between Gender, Age, and Incidence of Haematomas

Fifty-six per cent of the participants with HMTs were in the 60–79 age group. However, the distribution varied depending on gender. Not only were HMTs more frequent in women, but more than 71% of women with HMTs were in the age range 60–79. For men, the age range with the highest percentage of participants with HMTs was 40–59, with more than 57%. Despite the difference in these percentages, there are no significant differences in the incidence of MHTs across age groups.

While 84.7% of the sample presented HMTs, if the HMT distribution is analysed according to gender, almost twice the percentage of participants with HMTs were women compared to men (62.4% vs. 37.4%). Nonetheless, no significant differences were found. The observed differences in HMT incidence between genders (in our study) agree with some previously published results [5,8,53]. Chan [5] also concluded that women have a 7.5–10-fold increased risk of developing HMTs compared to men due to oestrogen levels and the strength of the capillaries. If oestrogen levels decrease in women, it causes a decrease in collagen and elasticity and an increase in the fragility of the capillaries [34]. Other authors [14,15] did not find a statistically significant correlation between these variables in their studies. These variations in the results of different studies suggest that we should continue including the variable gender in future studies, since according to some authors [40], oestrogen causes more vascularisation in women, which leads to greater bleeding at the injured injection site.

### 4.4. Obesity and Haematomas

Regarding the patient characteristics that were obtained in this study, such as weight and height, they cannot be compared with the annual data of the admission unit where the study was carried out, since participants are not weighed or measured during admission. Rather, the illustrative data shown in the medical record are revealed by the patient to the nurse during the interview at admission and to the anaesthetist before surgery.

The average BMI of the sample was 28.81 kg/m^2^, which is considered overweight. It is noteworthy that 42.5% of the sample was in the obese range (>30 kg/m^2^), a result that differs greatly from the prevalence (17.4%) of obesity in Spain [35] for the population over 18 years. However, if we focus on the population older than 65 years, the prevalence of obesity is 38.5%, therefore, our average is closer to the weighted average of the prevalence of obesity in the elderly [35]. If we take into account the average of the variables abdominal perimeter (100.35 cm) and ASF (32.00 mm) together with the BMI, close to 44% of the sample is obese, which represents a very high percentage by itself, and is close to the value shown in the BMI (42.5%).

### 4.5. Abdominal Skinfolds, Incidence of Appearance of Haematomas, and Area of Haematomas in Participants Receiving Low-Molecular-Weight Heparin

In our sample, individuals in the SF IV group presented more than 50% of the HMTs.

A significant relationship between ASF and the appearance of HMTs was found. A subject with an ASF over 36 mm has a 2.5 times greater risk of developing HMTs compared to individuals with a smaller SF. These results are confirmed with the AOR, which reveals a 1.8 times greater risk of such injuries. Thus, every time that the SF increases by 1 mm, the risk of developing HMTs increases by 4%. Like this study, Aguilera et al. [7] in their study with two obese groups (SF > 40 mm), found that not grabbing the abdominal pinch to administer the LMWH was positively correlated with the non-appearance of HMTs. This might suggest that the standard procedure for the administration of LMWH (with pinch) increases the appearance of HMTs in obese people, in line with our results. However, the authors of the previous study used a high SF cut-off value to distinguish between obese and non-obese individuals; thus, there might be obese individuals (those with an SF of 32–39 mm) in the non-obese group [54], which would introduce a bias behaviour into the study.

Regarding the ASF and the location of the HMTs, despite having found a correlation between them initially, after eliminating possible confounding variables, the relationship disappeared. No relationship was found between the ASF and the area of the HMTs.

### 4.6. Analysis of the Relationship between Body Mass Index and Incidence of Abdominal Haematomas

An individual with a BMI ≥ 25 kg/m^2^ (overweight/obesity) [52] presents a 65% greater risk of developing HMTs compared to an individual with a normal weight. This relationship is confirmed by eliminating possible confounding variables, although this results in a slightly lower increase in risk (56%). In contrast, Palese et al. [15] found that a high BMI (overweight/obesity) had a protective effect against the appearance of HMTs. However, in their discussion, these authors claimed that the lower incidence of HMTs observed in overweight/obese participants might be related to low doses of exposure to the drug according to the standard rule of dosage. They also attributed it to skin discolouration, which makes HMTs harder to detect in overweight/obese participants [5]. In addition, they affirmed that these results might be due to their sample having a high percentage of overweight/obese participants (40%). However, in our sample, the percentage of participants in this group (overweight/obese) was 72.2%. It is difficult to compare our results regarding these two variables with those of prior studies due to the lack of data in the literature on this topic.

Some authors [55] demonstrated that a single dose of enoxaparin for overweight/obese participants significantly decreases the prevalence of bleeding and HMTs. In addition, they claimed that the appearance of HMTs is a “biomarker” of greater bleeding. Moreover, some authors [38] affirmed that abdominal surgery in obese participants involves greater difficulties than those encountered in participants with a normal weight due to the precarious vascularisation of the thick layer of the cutaneous cellular tissue, which produces more trauma to the tissues. This suggests that an invasive technique such as an injection could produce more injuries in an obese individual.

In addition, obesity, diabetes, hypertension, and dyslipidaemia produce modifications in the walls of the vessels that increase their probability of breaking and releasing their contents, producing HMTs [56]. I believe that this might be a cause of the increase in HMTs. Studying these pathologies, evaluating the way they influence HMTs, and using the presence of HMTs as a “biomarker” to detect possible bleedings [52] could be of great interest for countries with a high incidence of these pathologies.

### 4.7. Relationship between Body Mass Index and the Area of the Abdominal Haematomas

In this study, the highest average area of HMTs is observed in obesity class II, followed by the overweight group. However, the obesity class II category comprised only 10 participants. As mentioned in the previous section, obese participants may suffer more injuries due to the thick layer of cellular tissue [38], and these injuries might be more serious. In addition, obesity produces modifications in the endothelium, including the anticoagulant and procoagulant capability, which could cause an increase in injuries after abdominal SC injection. Although we did not find a correlation between BMI and the area of the HMTs, it is necessary to continue researching this at-risk population due to the limited sample of the subgroup.

When comparing the size of the HMTs in this study with those reported in the existing literature, it is necessary to emphasise, firstly, that different terms are used in the studies to refer to the injuries (HMTs, bruises, contusions), and secondly, that the measurements carried out to determine the extent of the injuries are different. For example, Akpinar and Celebiogl [23] used the term contusions and measured them from a minimum linear diameter (0 mm), while Chan [5] and Zayback and Khorshid [9] each assessed the surface of the contusion in mm^2^, as was performed in this study.

Advantages and limitations: the study shows that obesity is a risk in the development of HMTs (both based on BMI and SF assessment), and that this risk remains when adjusted by removing confounding variables, finding that for every mm of ASF increases the risk of HMTs by 4%. Among the study’s limitations, the presence of other variables that could be modulating the appearance of HTs are not taken into account, such as the type of diet (vitamin B12, B9, C, or K deficiency), the patient’s own genetics, as pathologies such as diabetes or arterial hypertension were not included. However, if the absence of a previous diagnosis of coagulation disorders was verified, or if that the patient received another type of injection in the abdomen (e.g., insulin) or anticoagulant or antiaggregant treatment. The ASF measurement could be modified from day zero of uptake to the fifth day, after receiving the last dose of LMWH, and it was not measured every day so as not to stimulate the area before injection, or press with the lipocaliper after administration, promoting the increase in HMTs. It is necessary to continue looking for an answer. What ASF value should we use to look for a LMWH administration technique? Carrying out further studies with modified injection techniques for obese patients is required.

## 5. Conclusions

Regarding the objectives proposed, the following can be concluded: (a) the standard administration procedure for LMWH used by the nurses at the admission unit of the HUOTS produces a high incidence of abdominal HMTs, as 84.7% of participants developed HMTs after the administration of SC LMWH. The incidence decreases to 51% if it is assessed by injection. The variables that explain the incidence of the HMTs are the height of the participants, age, size of the ASF, and BMI. (b) Adults with high ASF (>36 mm; group IV) present a higher incidence of local HMTs. The risk of the appearance of HMTs for this group is 1.8 times greater than in the groups with lower ASF (≤III). If the ASF is seen as a continuous variable, the significant differences remain, establishing that for each mm increase in ASF, the risk of developing HMTs increases 4%. (c) No significant relationship is found between the ASF and the area of the HMTs that appear after the administration of SC LMWH when adjusted for age, gender, and height. Thus, the variables age, gender, and height act as confounding variables in the existing relationship between the ASF and the area of the HMTs. (d) The variable gender is not associated with the appearance of HMTs. Similarly, no significant relationship is found between age and the incidence of HMTs.

The study shows that obesity is a risk in the development of HMTs (both based on BMI and skinfold assessment), and that this risk remains when adjusted by removing confounding variables, finding that every mm of ASF increases the risk of HMTs by 4%. Among the limitations of the study is providing an answer: from what ASF value is it necessary to look for a LMWH administration technique? However, more studies are needed with modified techniques for obese patients.

The increase in obesity worldwide has been increasing, and this generates the need for further research (experimental studies) in order to find a LMWH administration technique for obese people that generates a lower number of HMTs, to avoid loss of adherence to treatment.

## Figures and Tables

**Table 1:** Variables.

Variables	Description
Age	Age of the subject in years
Gender	Male or female.
Height	Height of the subject in centimeters (cm).
Weight	Body weight of the subject in kilograms.
Body mass index (BMI)	It represents the relationship between weight in kilograms and height in square meters (kg/m^2^).
BMI category	Depending on the BMI, subject will be included in one of the six categories (according to the WHO): underweight (I; <18.5 kg/m^2^), normal weight (II; 18.5–24.9 kg/m^2^), overweight (III; 25–29.9 kg/m^2^), obesity class I (IV; 30–34.9 kg/m^2^), obesity class II (V; 35–39.9 kg/m^2^), obesity class III (VI; ≥40 kg/m^2^).
Dose of LMWH	Prescribed dose of Clexane in milligrams (mg).
Value of the ASF	Average value of the ASF in millimeters (mm) as a result of the average of three measurements: possible values 0–48 mm
ASF categories	Quartile of the ASF, which is obtained by dividing in four categories the possible values 1–48 mm of the abdominal ASF: ASF I (1–12), ASF II (12–24), ASF III (24–36), and ASF IV (36–48). Since just the measurement of the SF does not classify a subject as obese, the interpretation of the SF is performed according to the Likert scale: low, moderate, high, very high.
Administration area of LMWH in abdomen	Administration area of LMWH in abdomen: right hemiabdomen or left hemiabdomen. The nurse rotates the injection sites on the abdomen every day.
Clexane injection frequency	Every 24 h (four days)
HMT after the administration of LMWH	Presence of HMTs in the abdominal area 48 h after the injection of LMWH, possible values: yes or not.
Value of the HMT	Measurement of the HMT 48 h after the injection of LMWH. Value of length and width, both in mm.
Area of the HMT	Size of the HMT 48 h after the injection of LMWH. This value is obtained from the value of the HMT, as a result of the product of length and width, in square millimeters (mm^2^)

**Table 2:** Average and total area of HMTs for gender in the participants with HMTs.

HMTs	Total	Male (n = 64)	Female (n = 107)
Total area (mm^2^) of all participants with HMTs (n = 171)	125,569	41,406	84,163
Average area (mm^2^) per participants with HMTs (n = 171)	734.32	646.97	786.57
		Male (n = 139)	Female (n = 279)
Average area (mm^2^) HMTs (n = 418)	300.4	297.9	301.6

**Table 3:** Distribution of HMTs abdominal average area (mm^2^) by age (years) groups.

	30–39 (n = 7)	40–49 (n = 33)	50–59 (n = 18)	60–69 (n = 44)	70–79 (n = 46)	80–89 (n = 23)
Total area (mm^2^) of all participants with HMTs (n = 171)	8867	16,351	4820	31,557	53,072	10,902
Average area (mm^2^) per participants with HMT (n = 171)	1266.7	495.48	267.7	717.2	1153.7	474
	(n = 18)	(n = 77)	(n = 40)	(n = 117)	(n = 118)	(n = 48)
Average area (mm^2^) HMTs (n = 418)	492.6	212.3	120.5	269.7	449.7	227.1
	(n = 10)	(n = 39)	(n = 18)	(n 0 48)	(n = 52)	(n = 35)
Average area (mm^2^) HMTS (n = 202)	886.7	419.2	267.7	657.4	1020.6	311.4

**Table 4:** Total number of HMTs (n 418) according to BMI (obesity criteria WHO).

	N = 418		Male (n = 139)		Female (n = 279)	
	Frequency	Percent (%)	Frequency	Percent (%)	Frequency	Percent (%)
Underweight	-	-	-	-	-	-
Normal weight	98	23.4	3	2.2	95	34.1
Overweight	119	28.5	46	33.1	73	26.2
Type I obesity	168	40.2	78	56.1	90	32.3
Type II obesity	33	7.9	12	8.6	21	7.5
Extreme obesity	-	-	-	-	-	-

(-) Unavailable sample.

**Table 5:** Number of total HMTs (n 418) according to value of the ASF (for quartiles) and gender.

	n = 418		Male (n = 139)		Female (n = 279)	
Type of ASF	Frequency	Percent (%)	Frequency	Percent (%)	Frequency	Percent (%)
ASF I	-	-	-	-	-	-
ASF II	77	18.4	14	3.3	67	16.1
ASF III	106	25.4	27	6.5	82	19.7
ASF IV	235	56.2	98	23.4	130	31.2

(-) Unavailable sample.

**Table 6:** Distribution of abdominal HMT area (mm^2^) by type of abdominal SF (quartiles).

Area HMTs	ASF I (n = 0)	ASF II (n = 41)	ASF III (n = 48)	ASF IV (n = 82)
Total area (mm^2^) of all participants with HMTs (n = 171)	-	34.875	27.878	62.816
Average area (mm^2^) per participants with HMT (n = 171)	-	850.6	580.7	766
	-	(n = 77)	(n = 106)	(n = 235)
Average area (mm^2^) injury (n = 418)	-	452.9	263	267.2
Average area (mm^2^) for the total sample (n = 202)	-	697.5	489.0	705.6

(-) Unavailable sample.

**Table 7:** Regression model for logistics incidence of HMTs (crude and adjusted Odd Ratios).

Variables		Crude Odd Ratios			Adjusted Odd Ratios	
	OR	IC (95%)	P Wald	OR	IC (95%)	P Wald
Age	0.997	[0.99–1.01]	0.5423			
Gender						
Male	1					
Female	1.135	[0.81–1.59]	0.4573			
Weight	1.014	[1.01–1.03]	<0.005			
Size	0.993	[0.98–1.01]	0.3898			
ASF	1.045	[1.02–1.07]	<0.001	1.019	[0.995–1.043]	0.1151
Obesity levels–BMI (WHO)						
BMI II: normal weight	1			1		
BMI III: overweight	0.899	[0.57–1.43]	0.6552	0.93	[0.53–1.33]	0.456
BMI IV: type I obesity	1.65	[1.06–2.58]	<0.05	1.56	[1.05–2.32]	<0.05
BMI V: type II obesity	2.061	[0.801–5.3]	0.1335	2.23	[0.60–4.3]	0.639
ASF by categories						
ASF II (<24 mm)	1			1		
ASF III (24–36 mm)	1.224	[0.8–1.88]	0.3582	1.08	[0.7–1.68]	0.7207
ASF IV (>36 mm)	2.586	[1.71–3.92]	<0.001	1.8	[1.10–2.95]	<0.05

## Data Availability

The data that support the findings of this study will be available from the corresponding author, upon reasonable request.

## Abbreviations

Abdominal skinfold (ASF); adjusted odds ratios (AOR); body mass index (BMI); crude odds ratios (COR); electronic medical records (EMR); haematomas (HMTs); Hospital Insular de Gran Canaria (HIGC); hospital unit of orthopaedic and trauma surgery (HUOTS); low-molecular-weight heparin (LMWH); skinfold (SF); Spanish Agency for Medicines and Health Products (AEMPS); standard deviation (SD); subcutaneous (SC); strengthening the reporting of observational studies in epidemiology (STROBE); thromboembolic disease (TED); World Health Organization (WHO).
